# Massive Hemorrhage Associated With Upper Cervical Vertebral Fracture Treated Successfully With Transcatheter Arterial Embolization: A Case Report

**DOI:** 10.7759/cureus.51826

**Published:** 2024-01-07

**Authors:** Toshiro Imamoto, Makoto Sawano, Makoto Murase, Shinichi Yasuda, Tadashi Yahata

**Affiliations:** 1 Department of Emergency Medicine and Critical Care, Saitama Medical Center, Saitama Medical University, Kawagoe, JPN

**Keywords:** trauma induced coagulopathy, polytrauma patient resuscitation, cervical spine fracture, intervention radiology, blunt vertebral artery injury

## Abstract

Blunt vertebral artery injuries (BVAI) associated with cervical spine fractures are often problematic due to symptoms of occlusion. Denver grade V cases, in which the vertebral artery is transected, are rare but often fatal, and treatment has rarely been reported. We encountered a case of hemorrhagic shock due to an injury to a branch of the vertebral artery associated with an upper cervical spine fracture. Transcatheter arterial embolization was performed successfully to achieve hemostasis, requiring superselective arterial embolization to preserve the main trunk of the vertebral artery. It is important to be aware that vascular injuries to the branch vessels as well as the main trunk can cause complications.

## Introduction

Cervical spine trauma associated with blunt trauma with vertebral artery injury (VAI) is no longer rarely recognized, due to advances in imaging modalities [1]. In most cases, the problem is the symptoms of cerebral infarction caused by occlusion of the vertebral artery. As a result, treatment strategies for hemorrhage have rarely been addressed. We report a case of hemorrhagic and neurogenic shock due to VAI associated with cervical spine fracture, in which the main trunk of the vertebral artery was preserved by endovascular treatment, and hemostasis was achieved by embolization of only a branch of the artery.

## Case presentation

The patient was a 72-year-old male farmer who had been driving a tractor. A friend found the tractor overturned at the bottom of a bank, with a drop of about 10 m. The patient had been ejected from the vehicle. When the ambulance arrived, the patient was in cardiopulmonary arrest (CPA). Cardiopulmonary resuscitation was started immediately. Two minutes after patient contact, an airway was secured using a supraglottic device. One milligram of adrenaline was administered intravenously after securing the venous line. Spontaneous circulation returned 25 min after confirming CPA. The no-flow time was estimated to be up to 53 min and the low-flow time was estimated to be 25 min. During transport to the hospital, the patient showed bradycardia with a heart rate in the 30s and a drop in blood pressure to the point where the common femoral artery pulse was barely palpable, and noradrenaline and atropine had been administered by the time the patient arrived at the hospital. The patient was initially suspected of having neurogenic shock.

Vital signs on admission were: blood pressure, 111/68 mmHg; pulse rate, 115 beats/min; and respiratory rate, 14 times per minute regulated by a ventilator. Body temperature was 36.5°C. Glasgow Coma Scale (GCS) was 3 (E1VTM1; no eye-opening, no verbal response, no motion). Blood gas analysis at the time of admission indicated marked mixed acidosis: pH, 6.836; partial pressure of arterial carbon dioxide (PaCO2), 89.1 mmHg; partial pressure of arterial oxygen (PaO2), 137.9 mmHg; HCO3-, 15 mmol/L; lactate, 15 mmol/L.

Blood testing showed marked coagulopathy: fibrinogen level, <60 mg/dL; prothrombin time-international normalized ratio of prothrombin time (PT-INR), 2.14; and D-dimer, >400 μg/mL.

An arterial sheath was secured in the right femoral artery, a central venous channel was secured in the femoral vein, and an arterial line was secured in the left radial artery for monitoring. A massive transfusion protocol was then invoked. Type O blood transfusion was started and 3 g of fibrinogen concentrate was administered to rapidly correct the coagulopathy.

**Figure 1:**
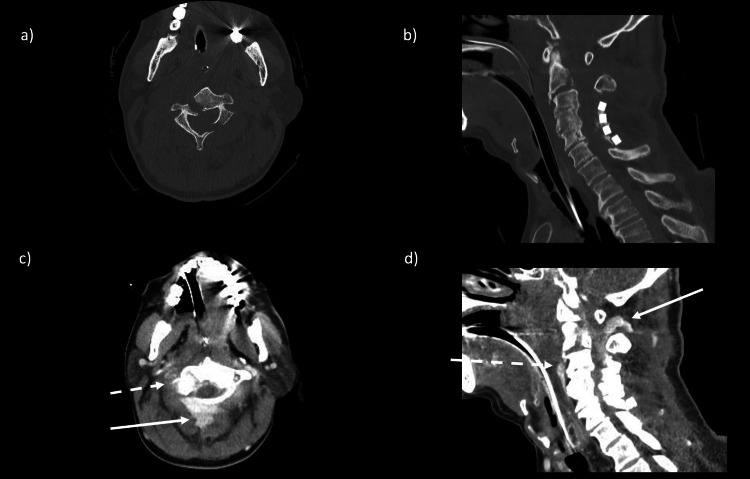
Plane CT neck in bone window algorithm, axial and sagittal views (a and b); enhanced CT neck in soft tissue algorithms, axial and sagittal views (c and d). a and b: Vertebral fracture of C2 and dislocation between C2 and C3 c and d: Marked extravascular leakage was seen on the anterior surface of the vertebral body (white dotted line), in the spinal canal of the cervical spine, and on the posterior surface of the vertebral body (white arrow).

Whole body contrast-enhanced computed tomography (CT) showed a vertebral fracture of C2 and dislocation between C2 and C3, with marked extravascular leakage at the posterior aspect of the vertebral body (Figure 1).

In addition, intramuscular hematoma with extravascular leakage was identified in the left buttock. The extravascular leakage in the left buttock was paler than in the cervical area.

Of course, once the patient was in CPA, coagulopathy was evident, and it was determined that each bleeding site needed to be treated with TAE to ensure hemostasis, but the amount of bleeding was clearly greater in the neck. The treatment strategy comprised manual repair of the cervical dislocation under fluoroscopic guidance, followed by endovascular treatment.

**Figure 2:**
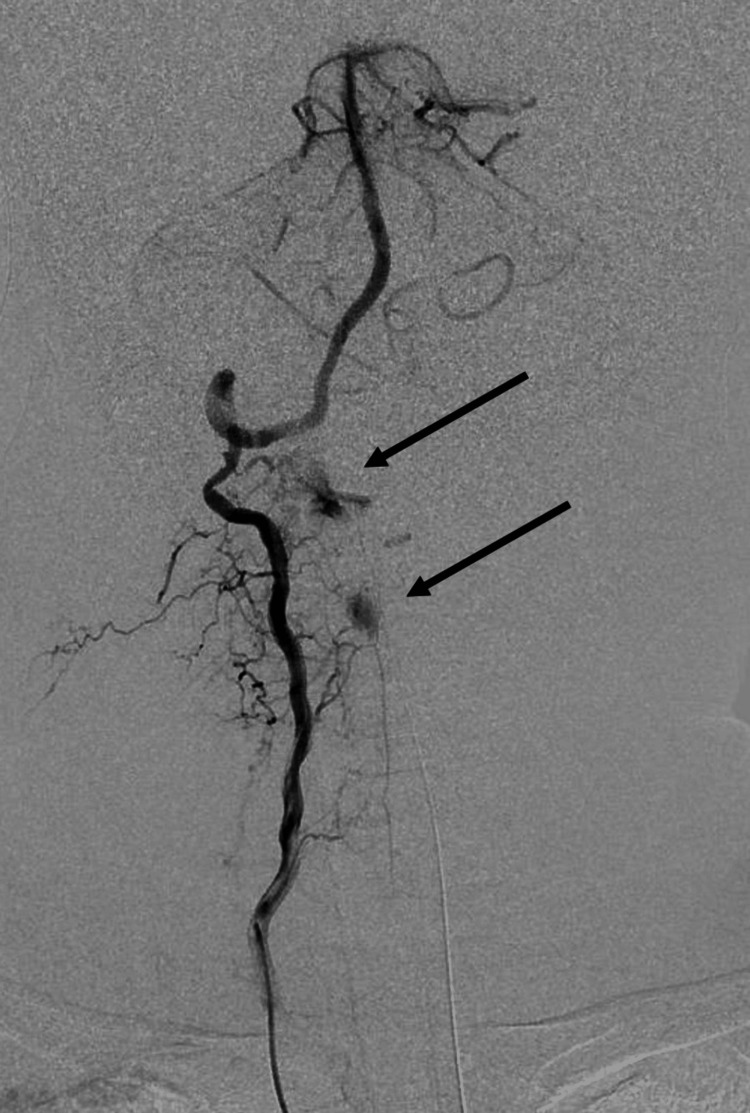
Angiography of the vertebral artery The main trunk of the vertebral artery is open, with no dissection. Two radicular arteries from V2 and V3 show significant extravascular leakage (black arrows).

Angiography was performed using a Cobra catheter to select the main trunk of the left internal iliac artery for embolization with a gelatin sponge fragment. Since the responsible vessel was difficult to identify on CT, aortography was performed using a pigtail catheter, revealing extravascular leakage from a branch of the right vertebral artery. The right vertebral artery was selected, and contrast was administered using an N2 catheter. The main trunk of the vertebral artery was open, with no dissection, but two radicular arteries from V2 and V3 showed significant extravascular leakage (Figure 2).

**Figure 3:**
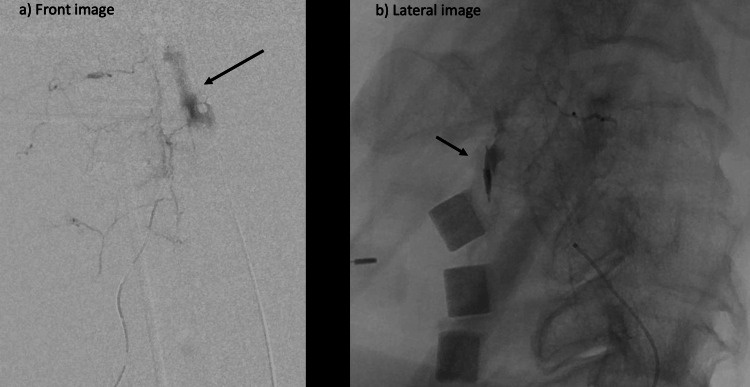
a/b Angiography of radicular artery (a) Coronal view; (b) Sagittal view. The sagittal view shows bleeding to the anterior surface of the vertebral body; this is caused by extravascular leakage from the radicular artery (black arrows).

We changed to a triple coaxial system to select the responsible vessel. The first branch could be selected, and the contrast showed marked and continuous extravascular leakage. Imaging during contrast infusion was performed in sagittal view and bleeding to the anterior surface of the vertebral body was consistent with extravascular leakage seen on the CT image (Figure 3).

**Figure 4:**
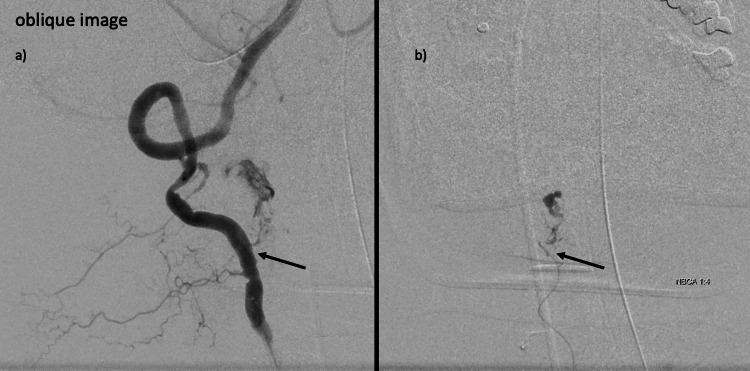
Angiography of another radicular artery a) Additional oblique imaging to identify the remaining radicular artery more clearly. Changing to a smaller-diameter catheter allows selection of the responsible vessel. The oblique view of the origin of the radicular artery makes it easier to visualize and catheterize the artery for selection. b) This vessel was similarly embolized using lipiodol + NBCA (4:1).

The same site was embolized using lipiodol + n-butyl-2-cyanoacrylate (NBCA). Extravascular leakage at the site disappeared on imaging. However, another extravascular leak remained. Selection of the remaining branches was considered difficult without the use of a smaller-diameter microcatheter and micro-guidewire, so an ev3 Marathon [microcatheter] (tip: 1.5 Fr; front: 2.7 Fr) and Traxcess14 [micro-guidewire] (tip: 0.012 inches; front: 0.014 inches) were used. Additional oblique imaging was used to identify the involved branches more clearly (Figure 4a). After these changes, vessel selection was performed. The branch vessel was similarly embolized using lipiodol + NBCA (Figure 4b).

**Figure 5:**
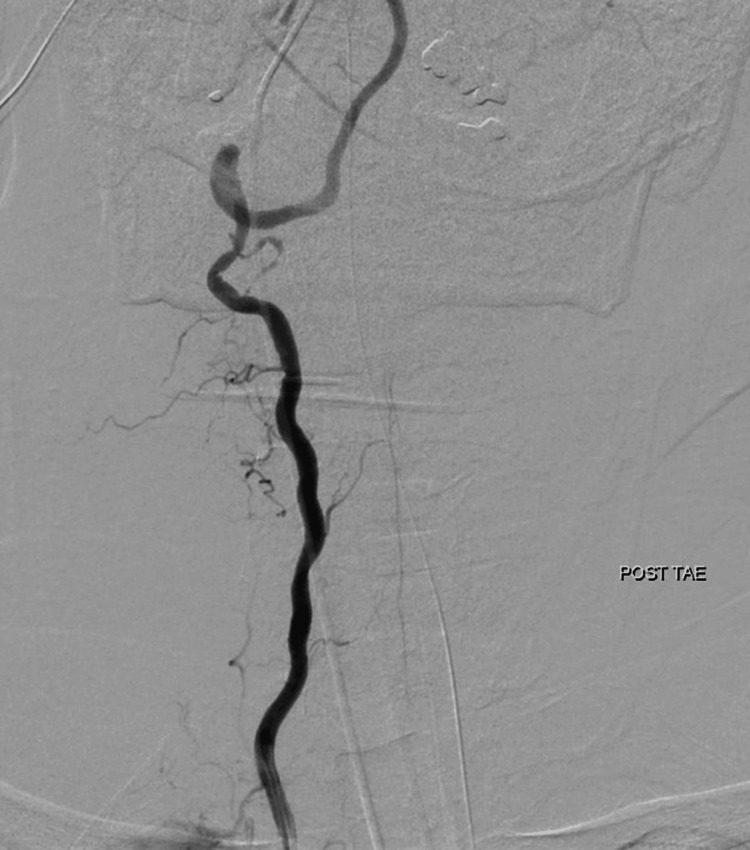
Angiography of the vertebral artery after TAE Extravascular leakage at the relevant site has disappeared.

Extravascular leakage at the relevant site disappeared on imaging (Figure 5).

**Figure 6:**
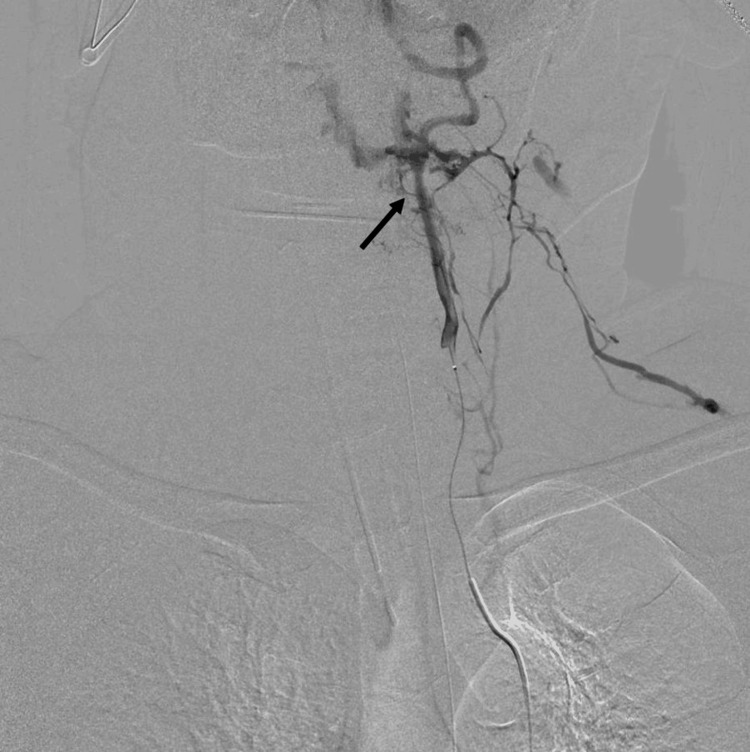
Angiography of the left vertebral artery Traumatic arteriovenous fistula is present, but no extravascular leakage is evident.

Angiography of the left vertebral artery revealed a traumatic arteriovenous fistula (AVF) without extravascular leakage (Figure 6).

Since hemostasis had been achieved, the treatment of the patient was ended without further action. After embolization, the patient was admitted to the ICU with reduced boosting agents.

Transfusion products administered within the first 24 hours after admission were: red blood cells, 14 U (1960 ml); fresh frozen plasma, 24 U (3360 ml); fibrinogen product, 6 g; Factor XIII, 720 U (15 U/kg); and four-factor prothrombin complex concentrate, 1500 U (25 U/kg). Coagulation system markers during treatment showed rapid correction with the use of coagulation factor preparations (Table 1).

**Table 1:** Changes in coagulation markers during initial treatment At the time of presentation, fibrinogen levels were very low, and PT-INR was prolonged. Aggressive administration of a coagulation factor preparation rapidly corrected the marked traumatic coagulopathy.

	Arrival at hospital	1 h after admission	2 h after admission	3 h after admission	4 h after admission	5 h after admission
PT (%)	19.9	36.6	28.9	12.9	11.9	11.5
PT-INR	2.14	3.78	3.03	1.43	1.32	1.28
APTT (sec)	174	174	174	46.5	39.1	50.6
D-dimer (μg/mL)	400	400	400	400	362	325
Fibrinogen (mg/dL)	60	60	60	110	147	223
Hb (g/dL)	11.4	8.8	13.5	9.3	9.9	10.1
Plt (×1000/μL）	190	192	176	135	93	105

The patient was severely affected by hypoxic encephalopathy after CPA and died on hospital Day 7 due to cerebral edema.

## Discussion

Our case suggests two points. First, BVAIs associated with cervical spine fractures rarely result in cases of major hemorrhage. Second, the patient was able to be treated with preservation of the main stem of the vertebral artery. In the past, VAI itself was considered a rare condition with an overall incidence of 0.2-0.77% among cases of blunt trauma [2].

With recent advances in radiological imaging and aggressive screening, the incidence of blunt trauma to the cervical spine has come to be viewed as a relatively frequent condition with an incidence of 17.2-22.5% among cases of blunt trauma [3].

VAI is often asymptomatic, but symptomatic cases often reportedly show sudden exacerbation of symptoms, including infarction of the posterior circulation and steal phenomenon [4]. The Denver grading scale is a morphological classification of injured vessels with regard to VAI: Grade I reflects an abnormal vessel wall, intramural hematoma or dissection with stenosis less than 25%; Grade II shows intramural hematoma or dissection with an embolus or dissected intima visible with stenosis of 25% or more; Grade III is pseudoaneurysm; Grade IV is complete vessel occlusion; and Grade V is dissection of the vessel or AVF [5]. VAI can also be classified by the site of origin: V1 is from the origin of the subclavian artery to the C6 foramen transversum; V2 is from the C6 foramen transversum to the C2 foramen transversum; V3 is from the C2 foramen transversum to the perforation of the dura mater at the foramen magnum occipitalis; and V4 is intradural [6]. In adults, 60% of cases are classified as V2. In the present case, multiple vessel injuries were observed in V2 and V3 and were somewhat extensive. The morphology of this case was not stenosis or obstruction, and the injury could be described as a dissection of a branch rather than a main trunk injury, which is primarily characterized by hemorrhage. This is an injury that did not match any of the traditional Denver grades. Such injuries are common in trunk trauma. A similar case of retropharyngeal hematoma requiring vertebral artery embolization has been reported, indicating that BVAI can be fatal not only via embolic symptoms but also through hemorrhagic symptoms [7].

In cases of obstruction or stenosis, VAI should be treated before cervical spine injury, and many concerns have been raised about cerebral infarction triggered by VAI. On the other hand, since Grade V is fatal, the likelihood of treatment being indicated is extremely low [8]. Surgical or endovascular treatment is the first choice for the treatment of grade V BVAI patients who arrive with signs of life [9]. In most cases, the main trunk is embolized or a peripheral stent graft is placed. Rarely, the main trunk of the vertebral artery is preserved via superselective embolization of a branch of the vertebral artery, as in the present case.

Circulation in the vertebrobasilar artery system can be preserved without preservation of the main trunk in two situations. One occurs with retrograde blood flow from the contralateral vertebral artery through the arterial circle of Willis. The other is when sufficient blood flows to the same site from ipsilateral collateral vessels, such as the thyroid carotid artery [10]. In the present case, the contralateral vertebral artery had a traumatic AVF, so retrograde blood flow could not be expected. Embolization that would preserve the main trunk was therefore chosen.

When cervical spine trauma is recognized, it is important to be alert to finding BVAI by carefully assessing contrast-enhanced CT and magnetic resonance angiography during the evaluation of cervical spine injuries and to be aware that BVAI is more common than assumed.

In addition, BVAI can be fatal as a main trunk injury or can lead to massive hemorrhage due to bleeding from branch vessels; therefore, trauma physicians must be familiar with treatment strategies in either circumstance.

## Conclusions

It should be remembered that blunt vertebral artery injuries rarely cause hemorrhagic shock, although in most cases, cerebral infarction is a problem for the patient.

Treatment of hemorrhagic vertebral artery injuries includes endovascular branch embolization, main stem embolization, and peripheral vascular stent grafting.

It is essential to evaluate whether cerebral circulation can be maintained by the contralateral normal vertebral artery or collateral circulation from the ipsilateral cervical vessels, to determine whether the main trunk can be preserved, and to be familiar with which treatment option to choose.
